# Clinical and gustatory features in taste disorder patients based on oral *Candida* culture status

**DOI:** 10.1007/s00784-026-06816-9

**Published:** 2026-03-13

**Authors:** Sung Min Kim, Hong-Seop Kho

**Affiliations:** 1 Department of Oral Medicine and Oral Diagnosis, School of Dentistry and Dental Research Institute, Seoul National University, 101, Daehak-ro, Jongno-gu, Seoul, 03080 South Korea; 2 Institute on Aging, Seoul National University, Seoul, 03080 South Korea

**Keywords:** Burning mouth syndrome, Candida, Dysgeusia, Hypogeusia, Hyposalivation, Taste disorders

## Abstract

**Objectives:**

To investigate the clinical, gustatory, salivary, psychological, and laboratory features of patients with taste disturbances, stratified by burning mouth syndrome (BMS) and *Candida* status, and to determine the contribution of oral *Candida* status independent of BMS subtype.

**Materials and methods:**

This retrospective study analyzed 141 patients with taste disturbances, classified into six groups based on burning mouth symptoms and oral *Candida* culture status: primary BMS, secondary BMS, and non-BMS patients, each subdivided by *Candida* status. To evaluate the independent role of *Candida*, positive *Candida* culture was not considered a local factor in defining secondary BMS. Clinical characteristics, taste test results, whole salivary flow rates, psychological profiles, and blood laboratory parameters were compared across groups, with age-matching applied when groups differed in age.

**Results:**

In the primary BMS, *Candida*-positive patients showed no normogeusia and had significantly lower objective taste scores after age-matching. Stimulated whole salivary flow rates were significantly reduced in *Candida*-positive patients with primary BMS and in those without burning mouth symptoms. Psychological distress was more pronounced in the *Candida*-positive subgroups within the primary BMS and in overall BMS groups.

**Conclusions:**

A positive oral *Candida* culture is not universally associated with impaired taste but is associated with gustatory deficits, hyposalivation, and psychological stress in specific subgroups, particularly primary BMS.

**Clinical relevance:**

A positive oral *Candida* culture may aggravate oral symptoms and psychological distress in patients with BMS, particularly those with primary BMS. Recognizing *Candida* as an independent variable could improve etiology-based diagnosis and tailored management of taste-related complaints.

**Supplementary Information:**

The online version contains supplementary material available at 10.1007/s00784-026-06816-9.

## Introduction

Taste disturbances are increasingly reported in dental practice and may impair appetite, reduce dietary intake, and diminish quality of life [1, 2]. Their prevalence increases with age due to physiological decline in gustatory function and the cumulative burden of multimorbidity, polypharmacy, and oral health problems in older adults [1, 3], leading to changes in dietary habits, nutritional compromise, and greater physical frailty risk [4]. Etiologies are multifactorial, including aging-related degeneration, local or systemic diseases, medication use, salivary gland hypofunction, and psychological conditions such as depression or anxiety [1, 5]. Aging-related degeneration may involve structural and functional changes in taste buds, peripheral gustatory nerves, and salivary gland function, which together contribute to reduced taste sensitivity in older adults [6]. Clinically, taste disturbances are evaluated using a combination of subjective symptom assessment and objective testing methods, including psychophysical taste tests (e.g., taste strips or whole-mouth testing), salivary flow rate measurements, and laboratory or microbiological investigations when indicated. However, diagnosis is further complicated by discrepancies between self-reported symptoms and objective test results, as well as variability in psychophysical testing methods [3, 7].

Burning mouth syndrome (BMS) is a chronic orofacial pain disorder characterized by persistent intraoral burning or dysesthetic sensations without visible mucosal abnormalities, often accompanied by dysgeusia and xerostomia [8–10]. It is classified as primary BMS when no cause is identified, and secondary BMS when symptoms relate to local or systemic factors such as candidiasis, anemia, or nutritional deficiencies [11]. Differentiation remains challenging due to overlapping symptoms and a lack of standardized criteria [12, 13]. Taste disturbances are among the most frequent BMS-associated symptoms and may involve neuropathic mechanisms, including altered taste–pain interactions. These complexities highlight the need for diagnostic approaches that consider the diverse mechanisms underlying both burning symptoms and taste disturbances [14, 15].

Oral *Candida* infection is common in patients with oral burning sensations, xerostomia, or taste disturbances, particularly among older adults [16, 17], and has been linked to mucosal irritation, reduced salivary function, and nutritional deficiencies [18]. Although often considered a contributor to oral discomfort and taste disturbances, its role remains controversial. Some studies have suggested that even without overt candidiasis, colonization can cause subclinical mucosal changes leading to oral pain or dysgeusia, which improves following antifungal therapy [19, 20]. Others, however, report inconsistent findings [21].

Given these inconsistencies, further research is needed to clarify the independent role of *Candida* in taste disturbances. Prior studies have been limited by inadequate patient stratification according to burning symptoms or *Candida* status, as well as by insufficient control for confounders such as age and systemic health. Furthermore, few studies have simultaneously evaluated subjective and objective taste measures, salivary flow rates, psychological profiles, and laboratory data. These limitations have hindered a clearer understanding of role that *Candida* plays in taste disorders and related symptoms.

To address these gaps, this retrospective study classified patients with taste disturbances according to the presence or absence of burning mouth symptoms and oral *Candida* culture status. To specifically examine the independent effects of *Candida*, a positive *Candida* culture was deliberately excluded as a criterion for secondary BMS. This classification strategy enabled a clearer analysis of how oral *Candida* culture status influences taste function. This study aimed to investigate the effects of *Candida* on taste and related symptoms in patients with and without BMS.

## Materials and methods

This retrospective study analyzed electronic medical records of patients who presented to the Department of Oral Medicine, Seoul National University Dental Hospital, between June 1, 2018 and May 31, 2025 for evaluation of taste disturbances. The study protocol adhered to the Declaration of Helsinki and was approved by the Institutional Review Board (IRB) of Seoul National University School of Dentistry (IRB No. S-D20250010; approved on July 8, 2025). Owing to the retrospective design, the IRB waived the requirement for informed consent.

### Inclusion and exclusion criteria

Eligible patients were those who reported taste disturbances as their chief complaint and underwent comprehensive clinical evaluation, including oral *Candida* culture, quantitative gustatory testing using filter paper strips and salivary flow rate measurement. Patients who were under 18 years of age, pregnant, had significant communication or comprehension difficulties, or were unable to reliably complete the study questionnaires were excluded.

### Examination procedures

All patients underwent comprehensive assessments, including oral *Candida* culture, gustatory testing, salivary flow rate measurement, structured questionnaires on taste disturbances and burning mouth symptoms, psychological evaluation using the Symptom Checklist-90-Revised (SCL-90-R), and laboratory blood tests. Detailed procedures are described in the following sections.

### Oral Candida culture

Oral *Candida* culture status was assessed using a *Candida Detector* (Kamemizu Chemical Ind. Co., Ltd., Osaka, Japan), according to the manufacturer’s instructions. This system is designed as a semi-quantitative, culture-based screening tool for clinical evaluation of oral *Candida* presence. A swab sample was collected from the dorsum of the tongue, cultured at 37 °C for 48 h, after which colony growth was assessed based on predefined density categories. According to the manufacturer’s interpretation guidelines, a growth level corresponding to ≥ 10³ CFU/mL was considered indicative of positive *Candida* culture warranting clinical evaluation. Oral mucosal examination was routinely performed at the initial visit to assess clinical signs suggestive of oral candidiasis; however, for the purpose of this study, subgroup classification was based on culture results. When clinical findings were suggestive of oral candidiasis, patients received routine clinical management, including topical or oral antifungal therapy when indicated.

### Taste function test

Taste function was assessed using filter paper strips impregnated with tastants (*Taste Strips*; Burghart Messtechnik, Holm, Pinneberg, Germany) following the manufacturer’s instructions [22]. Each strip, with a tip area of 2 cm², was placed on the midline of the anterior tongue. A total of 18 strips were administered in a pseudo-random order per patient: four concentrations each of sweet (sucrose: 0.4, 0.2, 0.1, and 0.05 g/mL), salty (sodium chloride: 0.25, 0.1, 0.04, and 0.016 g/mL), sour (citric acid: 0.3, 0.165, 0.09, and 0.05 g/mL), and bitter (quinine hydrochloride: 0.006, 0.0024, 0.0009, and 0.0004 g/mL) tastes, plus two blanks.

After each application, the patients identified the perceived taste quality from predefined options (sweet, salty, sour, bitter, or no taste) and rinsed their mouth with water before placing the next strip. Each correct response to the 16 tastant strips was scored one point, resulting in a maximum possible score of 16. The two blank strips were not included in the scoring and were used solely for quality control to assess false-positive responses, in accordance with the original taste strip protocol [23]. Hypogeusia was defined as a total score below nine, whereas dysgeusia was defined as incorrect responses at the strongest or second-strongest concentration for any taste quality.

### Salivary flow rate measurement

Whole salivary flow rates were measured under unstimulated and stimulated conditions. Unstimulated whole saliva (UWS) was collected into polypropylene tubes using the spitting method over 10 min. For stimulated whole saliva (SWS), patients chewed paraffin wax for 2 min and swallowed the initial saliva; chewing continued, and saliva was collected for 5 min. The salivary flow rates are expressed in mL/min.

### Questionnaire for the subjective assessment of taste disturbances

All patients completed a structured questionnaire assessing the factors potentially related to taste disturbances [15]. The questionnaire included demographic and medical history, olfactory symptoms, and taste-related complaints.

Subjective taste function was also evaluated using the following predefined statements: ‘I can detect sweetness in cocoa, cakes, or candies’; ‘I can detect salt in chips or salted nuts’; ‘I can detect sourness in vinegar, pickles, or lemon’; and ‘I can detect bitterness in coffee, beer, or tonic water’. The responses were scored as follows: 2 points for ‘recognize easily’, 1 point for ‘recognize somewhat’, and 0 points for ‘recognize not at all’, with total scores ranging from 0 to 8.

Patients further selected descriptors best reflecting the type of taste disturbance they experienced: distorted taste (dysgeusia), phantom taste (phantogeusia), exaggerated taste (hypergeusia), reduced taste (hypogeusia), absent taste (ageusia), or normal taste (normogeusia). For classification, patients reporting dysgeusia, hypergeusia, and phantogeusia were grouped as having subjective dysgeusia; those reporting hypogeusia and ageusia were classified as having subjective hypogeusia and ageusia, respectively; and those reporting normal taste sensation were grouped as having subjective normogeusia.

### Questionnaire on burning mouth symptoms

Patients reporting burning mouth symptoms completed a structured questionnaire adapted from previous studies [24]. The questionnaire assessed the characteristics and severity of their symptoms, including burning, aching, stinging, numbness, taste disturbance, xerostomia, and the effect of oral complaints on daily life (Eff-life). Each symptom was rated using a visual analogue scale (VAS) ranging from 0 (no symptom) to 10 (most extreme symptom imaginable), and both the average and maximum intensities were recorded.

### Psychological distress evaluation

Psychological distress was assessed using the SCL-90-R [25], a 90-item self-report questionnaire in which each item is rated on a 5-point Likert scale ranging from 0 (“not at all”) to 4 (“extremely”), with higher scores indicating greater psychological distress. For analysis, standardized T-scores were used for the intergroup comparisons of nine symptom dimensions—Somatization, Obsessive–Compulsive, Interpersonal Sensitivity, Depression, Anxiety, Hostility, Phobic Anxiety, Paranoid Ideation, and Psychoticism—as well as for the three global indices: the Global Severity Index (GSI), Positive Symptom Distress Index, and Positive Symptom Total (PST).

### Laboratory blood tests

Laboratory blood tests were performed as part of the routine clinical work-up for patients presenting with taste disturbances and/or burning mouth symptoms. The tests included complete blood count, erythrocyte sedimentation rate, blood chemistry, serum iron, ferritin, magnesium, zinc, vitamin B_12_, folate, and thyroid function tests (T3, free T4, and thyroid stimulating hormone).

### Patient classification

**Fig. 1:**
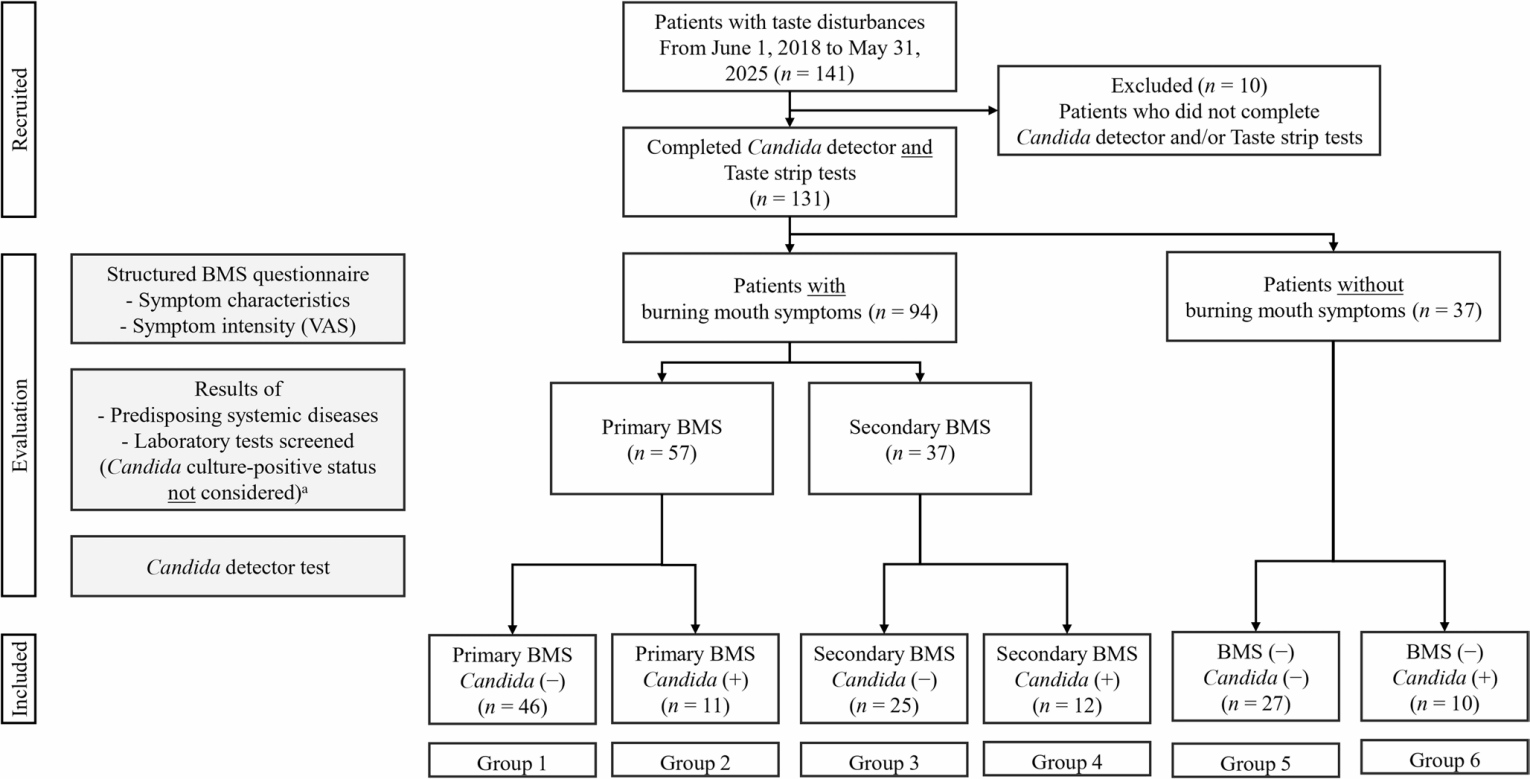
Flow diagram of patient recruitment, evaluation, and classification. Patients were categorized by the presence or absence of burning mouth symptoms and oral *Candida* culture status. Among those with burning mouth symptoms, classification into primary or secondary burning mouth syndrome (BMS) was determined by the presence of identifiable systemic abnormalities or local contributing factors. Group allocation (Groups 1–6) was based on the combination of BMS and oral *Candida* culture status for subsequent analyses ^a^ A positive oral *Candida* culture was not considered a local contributing factor for secondary BMS, aligning with the study’s aim of evaluating its influence independently

Figure 1 illustrates the patient inclusion, evaluation, and grouping process. Between June 1, 2018, and May 31, 2025, 141 patients visited the clinic for taste disturbances. Ten patients were excluded for incomplete diagnostic data, including missing *Candida* culture or taste strip test results. The final analysis included 131 patients (25 males, 63.0 ± 12.8 years; 106 females, 63.0 ± 11.0 years) who completed both oral *Candida* culture and taste function testing.

Of these, 94 patients reported burning mouth symptoms and were classified as having BMS based on their responses to the structured burning mouth symptom questionnaire; the remaining 37 reported no such symptoms. Patients with BMS were further subclassified as primary or secondary BMS based on the presence of identifiable systemic abnormalities or local contributing factors identified on clinical oral examinations, excluding positive oral *Candida* culture. Oral *Candida* culture-positive status was deliberately excluded as a contributing local factor for defining secondary BMS because the study aimed to evaluate its independent association with taste function regardless of BMS subtype. Accordingly, some *Candida*-positive patients without other explanatory conditions were classified as having primary BMS.

In *Candida*-positive BMS patients, classification into primary or secondary BMS was further supported by treatment response. In both subgroups, symptoms did not resolve with antifungal therapy alone, and neuropathic pain management was required. In secondary BMS, positive *Candida* culture coexisted with other identifiable systemic or local abnormalities linked to burning mouth symptoms, whereas in primary BMS, no such factors were identified despite persistent symptoms.

Subsequently, patients were categorized into six groups based on burning mouth symptoms and *Candida* culture results as follows: Group 1, primary BMS with *Candida*-negative results; Group 2, primary BMS with *Candida*-positive results; Group 3, secondary BMS with *Candida*-negative results; Group 4, secondary BMS with *Candida*-positive results; Group 5, non-BMS with *Candida*-negative results; and Group 6, non-BMS with *Candida*-positive results. These six groups were used for all subsequent analyses.

### Statistical analysis

Data normality was tested using the Shapiro–Wilk test. Depending on distribution, continuous variables between groups were compared using either Student’s t-test or the Mann–Whitney U test, whereas categorical variables were compared using the chi-square or Fisher’s exact tests, as appropriate.

When significant age differences were observed between the *Candida*-negative and *Candida*-positive subgroups, age-matching was performed to control for confounding factors. Specifically, the older half of the *Candida*-negative group was selected to match the age distribution of the *Candida*-positive group within each relevant comparison. All statistical analyses were conducted using IBM SPSS Statistics version 29.0 (IBM Corp., Armonk, NY, USA), with *p* < 0.05 considered statistically significant.

## Results

### Demographic characteristics and salivary flow rates

Table 1 summarizes the demographic characteristics and salivary flow rates across the six patient groups stratified by burning mouth symptoms and oral *Candida* culture status. Significant age differences were observed between the *Candida*-negative and *Candida*-positive subgroups within the primary BMS group (*p* = 0.009), with the *Candida*-positive patients being older. Similar age differences were also found in the total sample (*p* = 0.002) and in the overall burning mouth symptoms group (*p* = 0.010) (Supplementary Tables 1–1). Sex distribution did not differ in any comparison.

**Table 1:** Demographic characteristics and salivary flow rates based on burning mouth symptoms and oral *Candida* culture status; mean ± SD, median [Q1–Q3], *n* (%)

	Primary BMS (*n* = 57)		*p*	Secondary BMS (*n* = 37)		*p*	BM symptoms (−) (*n* = 37)		*p*
	*C* (−) (*n* = 46)	*C* (+) (*n* = 11)		*C* (−) (*n* = 25)	*C* (+) (*n* = 12)		*C* (−) (*n* = 27)	*C* (+) (*n* = 10)	
Age (years)	59.6 ± 9.8	68.3 ± 8.2	0.009**	64.9 ± 10.6	67.5 ± 11.3	0.426	60.8 ± 13.9	68.9 ± 9.5	0.100
Gender									
Male	4 (8.7)	1 (9.1)	1.000	3 (12.0)	2 (16.7)	1.000	12 (44.4)	3 (30.0)	0.481
Female	42 (91.3)	10 (90.9)		22 (88.0)	10 (83.3)		15 (55.6)	7 (70.0)	
SFR (mL/min)									
UWS	0.26 [0.16–0.37]	0.15 [0.07–0.27]	0.241	0.12 [0.05–0.30]	0.14 [0.06–0.21]	0.871	0.19 [0.08–0.30]	0.14 [0.03–0.31]	0.421
SWS	1.32 [0.80–1.64]	0.81 [0.73–0.97]^a^	0.001**	1.07 [0.67–1.61]	1.29 [0.66–1.75]^a^	0.336	1.10 [0.67–1.83]^a^	0.81 [0.55–1.02]^a^	0.006**

^a^ Stimulated whole saliva could not be collected from two patients with primary BMS (oral *Candida* (+)), two with secondary BMS (oral *Candida* (+)), and three without BM symptoms (one oral *Candida* (−), two oral *Candida* (+)), due to inability to chew paraffin wax caused by missing posterior teeth

*P*-values were calculated using the Student’s *t*-test or Mann–Whitney *U* test for continuous variables, as appropriate, and the Fisher’s exact test for gender comparisons

*BM* burning mouth, *BMS* burning mouth syndrome, *C (−)* oral *Candida* culture negative, *C (+)* oral *Candida* culture positive, *Q1* 25th percentile, *Q3* 75th percentile, *SD* standard deviation, *SFR* salivary flow rate, *SWS* stimulated whole saliva, *UWS* unstimulated whole saliva

** *p* < 0.01

In the primary BMS group, *Candida*-positive patients exhibited significantly lower SWS flow rates than *Candida*-negative patients, which are the patients with true primary BMS (*p* = 0.001), and this remained significant after age-matching (*p* = 0.043; Supplementary Tables 1–2). Among patients without burning mouth symptoms, the *Candida*-positive subgroup also had significantly lower SWS flow rate than the *Candida*-negative subgroup (*p* = 0.006). The UWS flow rates did not differ significantly in any comparison.

### Subjective and objective taste function

Table 2 presents the subjective and objective taste scores across the six patient groups. No significant differences in total subjective or objective taste scores were observed between the *Candida*-negative and *Candida*-positive subgroups in any comparison, including the total sample and the burning mouth symptoms group (Supplementary Tables 2 − 1). Similarly, no significant differences were found for individual taste qualities—sweet, salty, sour, and bitter. However, in the age-matched analysis, primary BMS patients with positive *Candida* culture exhibited significantly lower objective total taste scores than *Candida*-negative patients (*p* = 0.049; Supplementary Tables 2–2).

**Table 2:** Subjective and objective taste scores based on burning mouth symptoms and oral *Candida* culture status; median [Q1–Q3]

	Primary BMS (*n* = 57)		*p*	Secondary BMS (*n* = 37)		*p*	BM symptoms (−) (*n* = 37)		*p*
	*C* (−) (*n* = 46)	*C* (+) (*n* = 11)		*C* (−) (*n* = 25)	*C* (+) (*n* = 12)		*C* (−) (*n* = 27)	*C* (+) (*n* = 10)	
Subjective taste									
Sweet	2.0 [1.0–2.0]	2.0 [1.0–2.0]	0.681	2.0 [1.0–2.0]	2.0 [1.0–2.0]	0.867	2.0 [1.0–2.0]	2.0 [1.8–2.0]	0.431
Sour	2.0 [1.8–2.0]	2.0 [2.0–2.0]	0.272	2.0 [1.0–2.0]	2.0 [1.3–2.0]	0.917	2.0 [1.0–2.0]	2.0 [1.8–2.0]	0.629
Salty	2.0 [1.0–2.0]	2.0 [1.0–2.0]	0.784	2.0 [1.0–2.0]	2.0 [1.0–2.0]	0.874	2.0 [1.0–2.0]	2.0 [2.0–2.0]	0.147
Bitter	2.0 [1.0–2.0]	2.0 [1.0–2.0]	0.823	2.0 [1.0–2.0]	1.5 [1.0–2.0]	0.274	2.0 [2.0–2.0]	2.0 [0.8–2.0]	0.379
Total	8.0 [5.5–8.0]	7.0 [7.0–8.0]	0.617	8.0 [4.0–8.0]	7.0 [4.0–8.0]	0.537	8.0 [5.0–8.0]	8.0 [5.8–8.0]	0.802
Objective taste									
Sweet	4.0 [2.8–4.0]	4.0 [3.0–4.0]	0.867	3.0 [2.0–4.0]	4.0 [3.0–4.0]	0.503	3.0 [1.0–4.0]	3.0 [0.0–4.0]	0.986
Sour	2.0 [1.8–3.0]	1.0 [1.0–2.0]	0.117	2.0 [0.5–3.0]	2.5 [1.3–3.0]	0.290	2.0 [1.0–2.0]	1.0 [0.0–2.3]	0.076
Salty	3.0 [2.0–4.0]	3.0 [3.0–3.0]	0.975	2.0 [1.0–3.5]	1.5 [0.3–3.0]	0.200	2.0 [1.0–3.0]	3.0 [1.8–4.0]	0.141
Bitter	3.0 [2.0–4.0]	3.0 [0.0–3.0]	0.086	3.0 [1.0–4.0]	3.5 [1.3–4.0]	0.695	2.0 [1.0–3.0]	3.0 [0.8–3.0]	0.874
Total	11.5 [9.0–13.0]	10.0 [8.0–12.0]	0.094	10.0 [7.0–13.0]	10.5 [7.3–13.0]	0.835	8.0 [6.0–11.0]	8.5 [5.5–12.0]	0.893

Subjective taste scores were obtained from a structured questionnaire in which patients rated their ability to detect each taste quality on a scale from 0 (not at all) to 2 (easily), yielding a maximum total score of 8. Objective taste scores were obtained using filter paper taste strips (*Taste Strips*; Burghart Messtechnik, Holm, Pinneberg, Germany), with each taste quality (sweet, salty, sour, and bitter) scored 0–4 based on correct identifications (maximum total score 16)

*P*-values were calculated using the Student’s *t*-test or the Mann–Whitney *U* test, as appropriate, for comparisons between *Candida* (+) and *Candida* (−) within each group

*BM* burning mouth, *BMS* burning mouth syndrome, *C *(−) oral *Candida* culture negative, *C* (+) oral *Candida* culture positive, *Q1 *25th percentile, *Q3* 75th percentile

### Distribution of taste diagnoses

Table 3 and Supplementary Tables 3−1 summarize the distribution of objective and subjective taste diagnoses across the patient groups. In the primary BMS group, the distribution of objective taste diagnoses differed significantly between the *Candida*-negative and *Candida*-positive subgroups (*p* = 0.018). Specifically, none of the *Candida*-positive patients were classified as normogeusia, whereas 39.1% of the *Candida*-negative patients were. This difference persisted after age-matching (*p* = 0.008; Supplementary Tables 3−2). No significant differences were observed in the distribution of subjective taste diagnoses in any of the groups.

**Table 3:** Distribution of subjective and objective taste diagnoses based on oral *Candida* culture status in patients with primary burning mouth syndrome, secondary burning mouth syndrome, and without burning mouth symptoms; *n* (%)

	Primary BMS (*n* = 57)		*p*	Secondary BMS (*n* = 37)		*p*	BM symptoms (−) (*n* = 37)		*p*
	*C* (−) (*n* = 46)	*C* (+) (*n* = 11)		*C* (−) (*n* = 25)	*C* (+) (*n* = 12)		*C* (−) (*n* = 27)	*C* (+) (*n* = 10)	
Subjective taste									
Normogeusia	5 (10.9)	2 (18.2)	0.377	4 (16.0)	2 (16.7)	0.949	2 (7.4)	2 (20.0)	0.484
Hypogeusia	13 (28.3)	6 (54.5)		11 (44.0)	6 (50.0)		13 (48.1)	6 (60.0)	
Dysgeusia	12 (26.1)	1 (9.1)		2 (8.0)	1 (8.3)		5 (18.5)	2 (20.0)	
Hypo + Dysgeusia	13 (28.3)	2 (18.2)		7 (28.0)	2 (16.7)		5 (18.5)	0 (0.0)	
Ageusia	3 (6.5)	0 (0.0)		1 (4.0)	1 (8.3)		2 (7.4)	0 (0.0)	
Objective taste									
Normogeusia	18 (39.1)	0 (0.0)	0.018*	5 (20.0)	3 (25.0)	1.000	4 (14.8)	1 (10.0)	1.000
Hypogeusia	3 (6.5)	0 (0.0)		2 (8.0)	0 (0.0)		2 (7.4)	1 (10.0)	
Dysgeusia	19 (41.3)	7 (63.6)		10 (40.0)	5 (41.7)		9 (33.3)	4 (40.0)	
Hypo + Dysgeusia	6 (13.0)	4 (36.4)		8 (32.0)	4 (33.3)		12 (44.4)	4 (40.0)	

Subjective taste diagnoses were based on questionnaire responses: normogeusia was defined as normal taste perception; hypogeusia as self-reported decreased taste; dysgeusia as distorted, exaggerated, or phantom taste sensations; and ageusia as complete taste loss. Objective taste diagnoses were based on taste strip test results: normogeusia was defined as a total score ≥ 9; hypogeusia as a total score < 9; dysgeusia as incorrect responses at the strongest or second-strongest concentration for any taste quality. Hypo + dysgeusia indicates patients meeting both criteria

*P*-values were calculated using the Chi-square test or the Fisher’s exact test, as appropriate, for comparisons between oral *Candida* (−) and oral *Candida* (+) within each group

*BM* burning mouth, *BMS* burning mouth syndrome, *C* (−) oral *Candida* culture negative, *C* (+) oral *Candida* culture positive

* *p* < 0.05

### Intensities of burning mouth symptoms

Table 4 compares the burning mouth symptom intensities between *Candida*-negative and *Candida*-positive subgroups within each BMS category. In the primary BMS group, *Candida*-positive patients reported significantly higher VAS scores for the average levels of burning (*p* = 0.045) and aching (*p* = 0.032) symptoms than *Candida*-negative patients. In the age-matched analysis (Supplementary Tables 4−1), these differences were no longer statistically significant. In contrast, stinging intensity was significantly higher in *Candida*-positive patients for both average (*p* = 0.037) and maximum (*p* = 0.030) VAS scores. No other symptom domains showed statistically significant differences in either analysis.

**Table 4:** Burning mouth symptom intensities based on burning mouth symptoms and oral *Candida* culture status; median [Q1–Q3]

Intensity (VAS)		Primary BMS (*n* = 57)		*p*	Secondary BMS (*n* = 37)		*p*	BMS (+) (*n* = 94)		*p*
		*C* (−) (*n* = 46)	*C* (+) (*n* = 11)		*C* (−) (*n* = 25)	*C* (+) (*n* = 12)		*C* (−) (*n* = 71)	*C* (+) (*n* = 23)	
Burning	AVG	3.5 [2.0–5.0]	5.0 [3.0–7.0]	0.045*	5.0 [2.0–7.0]	5.0 [3.3–5.0]	0.480	4.0 [2.0–6.0]	5.0 [3.0–7.0]	0.223
	MAX	7.0 [5.0–8.0]	8.0 [6.0–8.0]	0.351	8.0 [3.0–9.0]	7.0 [4.3–8.0]	0.599	7.0 [5.0–8.0]	7.0 [5.0–8.0]	0.734
Aching	AVG	2.0 [0.0–4.0]	5.0 [0.0–6.0]	0.032*	0.0 [0.0–6.5]	0.5 [0.0–3.8]	0.671	1.0 [0.0–5.0]	3.0 [0.0–5.0]	0.387
	MAX	3.5 [0.0–7.0]	7.0 [0.0–8.0]	0.254	1.0 [0.0–8.0]	2.0 [0.0–7.8]	0.849	2.0 [0.0–7.0]	6.0 [0.0–8.0]	0.563
Stinging	AVG	0.0 [0.0–2.0]	2.0 [0.0–7.0]	0.055	0.0 [0.0–4.5]	0.0 [0.0–2.5]	0.386	0.0 [0.0–3.0]	1.0 [0.0–5.0]	0.409
	MAX	0.0 [0.0–5.0]	7.0 [0.0–8.0]	0.052	0.0 [0.0–7.5]	0.5 [0.0–6.5]	0.834	0.0 [0.0–5.0]	3.0 [0.0–8.0]	0.208
Numbness	AVG	0.0 [0.0–3.0]	1.0 [0.0–5.0]	0.274	0.0 [0.0–0.5]	0.0 [0.0–4.8]	0.261	0.0 [0.0–2.0]	0.0 [0.0–5.0]	0.146
	MAX	0.0 [0.0–5.3]	1.0 [0.0–8.0]	0.349	0.0 [0.0–1.5]	0.0 [0.0–6.0]	0.310	0.0 [0.0–5.0]	0.0 [0.0–7.0]	0.220
Taste−dist	AVG	5.0 [2.0–7.0]	7.0 [3.0–7.5]	0.299	6.0 [3.5–8.0]	5.0 [4.0–8.5]	0.961	5.0 [2.0–8.0]	6.0 [4.0–7.5]	0.416
	MAX	8.0 [5.0–9.0]	8.0 [6.0–8.0]	0.910	8.0 [5.5–9.0]	7.0 [5.3–9.8]	0.793	8.0 [5.0–9.0]	8.0 [6.0–9.0]	0.834
Xerostomia	AVG	2.5 [0.8–5.0]	5.0 [3.0–7.0]	0.144	3.0 [1.0–5.0]	4.0 [3.3–6.8]	0.300	3.0 [1.0–5.0]	5.0 [3.0–7.0]	0.061
	MAX	5.0 [2.0–8.0]	8.0 [3.0–9.0]	0.206	5.0 [1.5–8.0]	6.0 [5.0–8.8]	0.510	5.0 [2.0–8.0]	7.0 [5.0–9.0]	0.172
Eff−life	AVG	5.0 [2.0–7.0]	5.0 [4.0–8.0]	0.530	6.0 [4.5–8.0]	5.0 [3.0–7.3]	0.331	5.0 [2.0–7.0]	5.0 [3.0–8.0]	0.898
	MAX	7.0 [4.8–9.0]	8.0 [5.0–9.0]	0.660	8.0 [6.5–9.0]	7.0 [4.3–9.8]	0.718	8.0 [5.0–9.0]	8.0 [5.0–9.0]	0.887

Symptom intensities were assessed using visual analogue scale (VAS) scores ranging from 0 (no symptom) to 10 (most extreme symptom imaginable). AVG and MAX represent average and maximum scores, respectively, for each symptom domain

*P*-values were calculated using the Mann–Whitney *U* test for comparisons between *Candida* (+) and *Candida* (−) within each group.

*AVG* average, *BMS* burning mouth syndrome, *C* (−) oral *Candida* culture negative, *C* (+) oral *Candida* culture positive, *Eff-life* effect of symptoms on daily life, *MAX* maximum, *Q1* 25th percentile, *Q3* 75th percentile, *Taste-dist* taste disturbance, *VAS* visual analogue scale

### Psychological profiles

Table 5 presents the psychological profiles derived from the SCL-90-R across the six patient groups. In the primary BMS group, the *Candida*-positive subgroup showed significantly higher *t*-scores for somatization (*p* = 0.010), anxiety (*p* = 0.042), psychoticism (*p* = 0.049), GSI (*p* = 0.044), and PST (*p* = 0.032) than the *Candida*-negative subgroup. However, none of these differences remained statistically significant in the age-matched analysis. In both the total sample and the overall BMS group, the *Candida*-positive subgroup demonstrated significantly higher phobic anxiety (*p* = 0.026 and 0.045, respectively), psychoticism (*p* = 0.015 and 0.029), and PST (*p* = 0.025 and 0.021) than the *Candida*-negative subgroup. Some of these findings persisted in the age-matched analysis. In the age-matched total sample, the *Candida*-positive subgroup showed significantly higher scores for interpersonal sensitivity (*p* = 0.036), psychoticism (*p* = 0.048), and PST (*p* = 0.037). In the age-matched overall BMS group, the *Candida*-positive subgroup continued to show significantly higher PST scores (*p* = 0.040) (Supplementary Tables 5−1 and 5−2).

**Table 5:** Psychological profiles based on burning mouth symptoms and oral *Candida* culture status; median [Q1–Q3]

t-score	Primary BMS (*n* = 56)^a^		*p*	Secondary BMS (*n* = 35)^a^		*p*	BM symptoms (−) (*n* = 27)^a^		*p*
	***C*** **(−) (*n*** **= 45)**	***C*** **(+) (*n*** **= 11)**		***C*** **(−) (*n*** **= 24)**	***C*** **(+) (*n*** **= 11)**		***C*** **(−) (*n*** **= 19)**	***C*** **(+) (*n*** **= 8)**	
Somatization	43.0 [38.0–47.5]	48.0 [45.0–51.0]	0.010*	49.5 [44.3–53.0]	45.0 [43.0–54.0]	0.645	41.0 [38.0–44.0]	42.5 [37.3–44.8]	0.593
O-C	41.0 [34.5–46.0]	45.0 [40.0–51.0]	0.090	44.5 [39.0–50.3]	43.0 [38.0–57.0]	0.560	40.0 [36.0–44.0]	40.5 [38.5–46.0]	0.937
I-S	41.0 [36.0–48.0]	43.0 [41.0–52.0]	0.091	43.0 [39.0–44.0]	44.0 [36.0–55.0]	0.345	39.0 [36.0–41.0]	42.0 [36.8–46.0]	0.251
Depression	43.0 [37.0–49.0]	46.0 [42.0–53.0]	0.088	48.0 [43.3–53.8]	44.0 [41.0–54.0]	0.760	40.0 [38.0–48.0]	45.5 [37.3–52.3]	0.650
Anxiety	41.0 [38.0–44.0]	44.0 [42.0–46.0]	0.042*	45.0 [41.3–51.3]	43.0 [38.0–49.0]	0.549	40.0 [37.0–46.0]	40.0 [39.3–47.5]	0.540
Hostility	42.0 [38.0–45.0]	45.0 [42.0–48.0]	0.074	43.0 [40.0–45.0]	43.0 [38.0–47.0]	0.801	40.0 [38.0–43.0]	40.0 [40.0–42.8]	0.785
Phobic anxiety	42.0 [40.0–45.0]	43.0 [40.0–48.0]	0.295	42.0 [40.0–45.0]	43.0 [42.0–56.0]	0.095	43.0 [40.0–45.0]	44.0 [42.0–47.3]	0.464
Paranoid ideation	40.0 [38.0–42.0]	40.0 [38.0–51.0]	0.378	40.0 [38.0–44.3]	40.0 [38.0–48.0]	0.546	38.0 [38.0–42.0]	40.0 [38.0–43.8]	0.405
Psychoticism	41.0 [39.0–45.0]	45.0 [41.0–50.0]	0.049*	44.0 [41.3–47.5]	45.0 [42.0–50.0]	0.164	41.0 [38.0–45.0]	43.5 [41.0–50.5]	0.238
GSI	42.0 [37.0–46.5]	44.0 [42.0–50.0]	0.044*	45.0 [42.0–49.8]	43.0 [39.0–58.0]	0.762	40.0 [37.0–44.0]	41.5 [37.5–47.0]	0.678
PSDI	41.0 [39.0–45.0]	41.0 [39.0–48.0]	0.975	47.0 [43.3–53.5]	43.0 [39.0–52.0]	0.151	44.0 [39.0–48.0]	44.5 [37.5–48.0]	0.937
PST	42.0 [33.5–48.0]	48.0 [42.0–57.0]	0.032*	46.0 [39.3–50.0]	46.0 [37.0–58.0]	0.442	40.0 [35.0–43.0]	44.0 [33.8–49.8]	0.238

Psychological profile scores are presented as *t*-scores from the Symptom Checklist-90-Revised (SCL-90-R), comprising nine symptom dimensions and three global indices

^a^Due to incomplete responses, SCL-90-R data were available for 56 of 57 primary BMS patients, 35 of 37 secondary BMS patients, and 27 of 37 patients without burning mouth symptoms

*P*-values were calculated using the Student’s *t*-test or the Mann–Whitney *U* test, as appropriate, for comparisons between *Candida* (+) and *Candida* (−) within each group

*BM* burning mouth, *BMS* burning mouth syndrome, *C* (−) oral *Candida* culture negative, *C* (+) oral *Candida* culture positive, *GSI* global severity index, *I-S* interpersonal sensitivity, *O-C* obsessive compulsive, *PSDI* positive symptom distress index, *PST* positive symptom total, *Q1* 25th percentile, *Q3* 75th percentile

* *p* < 0.05

### Laboratory blood test results

No statistically significant differences were observed between *Candida* subgroups within either the primary or secondary BMS groups (data not shown). Among patients without burning mouth symptoms, however, the *Candida*-positive subgroup showed significantly lower serum albumin (*p* = 0.030), and higher alkaline phosphatase (*p* = 0.039) levels than the *Candida*-negative subgroup. In the total sample, the *Candida*-positive group exhibited significantly lower total cholesterol level (*p* = 0.041). In the overall BMS group, the *Candida*-positive subgroup also showed lower eosinophil counts (*p* = 0.041) and lower total cholesterol (*p* = 0.028) and lower serum iron (*p* = 0.048) levels than the *Candida*-negative subgroup (data not shown).

In the age-matched analyses, the primary BMS group again showed no significant differences between *Candida* subgroups. However, the age-matched total sample revealed lower mean corpuscular volume (*p* = 0.021) in the *Candida*-positive group, whereas in the age-matched burning mouth symptoms group, the *Candida*-positive subgroup had significantly higher red blood cell counts (*p* = 0.046) than the *Candida*-negative group (data not shown).

## Discussion

This retrospective study examined the clinical, gustatory, salivary, psychological, and laboratory profiles of patients with taste disturbances based on burning mouth symptoms and culture-detected oral *Candida* status identified using a semi-quantitative screening assay, using a six-group classification. Unlike previous studies, we deliberately excluded *Candida* culture-positive status from the definition of secondary BMS, allowing us to isolate its potential independent contribution to taste-related outcomes. This approach enabled direct comparison of subgroups rarely distinguished in earlier work and provided new insights into how oral *Candida* culture-positive status may interact with neuropathic mechanisms in BMS to influence gustatory function. Notably, oral *Candida* culture-positive primary BMS patients displayed a distinctive gustatory profile warranting closer examination.

In this cohort, *Candida* culture-positive primary BMS patients exhibited no normogeusia and a significantly reduced objective taste score after age-matching, indicating a selective vulnerability of this subgroup to *Candida*-associated gustatory impairment. This vulnerability may reflect neuropathic alterations characteristic of primary BMS, including peripheral small-fiber dysfunction and altered taste–pain interactions, which create a sensitized baseline state [26–28]. Within this context, additional effects of oral *Candida* colonization may exceed functional thresholds and manifest as clinically apparent gustatory impairment [29–31].

Beyond peripheral mechanisms, a subset of primary BMS patients has been shown to exhibit central sensitization, characterized by amplified nociceptive processing and exaggerated responses to minor stimuli [32]. Within this peripherally and centrally sensitized trigeminal–gustatory network, oral *Candida* presence may further influence taste perception and intensify oral discomfort. Reports of taste improvement after antifungal therapy in some patients without overt candidiasis support the possibility of microbially mediated aggravation of pre-existing neural dysfunction [19, 20], although inconsistent findings across studies suggest that such effects may depend on the underlying neuropathic context of primary BMS [21]. Collectively, these findings support a potential interaction between neuropathic susceptibility and oral *Candida* colonization in shaping gustatory disturbances. These interpretations should be regarded as hypothesis-generating and based on observed associations rather than causal mechanisms, consistent with our finding of greater pain severity in *Candida*-positive patients.

Furthermore, *Candida*-positive patients with primary BMS and those without burning mouth symptoms exhibited significantly lower stimulated whole salivary flow rates, a difference that remained significant in the primary BMS subgroup after age-matching. This finding is consistent with meta-analytic evidence that hyposalivation increases the risk of oral *Candida* colonization or candidiasis by approximately threefold [33]. The relationship is likely bidirectional: reduced salivary flow impairs mechanical clearance, lowers the concentrations of antifungal proteins, including histatins, lactoferrin, and lysozyme, and reduces buffer capacity [34, 35], thereby facilitating *Candida* adhesion, hyphal transformation, and biofilm formation [16, 36].

Conversely, *Candida* infection itself may impair salivary gland function either through inflammatory involvement of the ductal epithelium or by altering local neural regulation of secretion, creating a vicious cycle of persistent colonization and salivary hypofunction [37]. Clinical data support this reciprocity, as antifungal therapy has been shown to significantly increase stimulated salivary flow rates and reduce xerostomia and tongue pain [38]. In our study, reduced stimulated salivary flow rates were consistently observed in *Candida*-positive patients, even in those without burning mouth symptoms, suggesting that salivary hypofunction predisposes to *Candida* colonization and that persistent infection, in turn, exacerbates salivary dysfunction, with potential implications for oral comfort, taste perception, and quality of life.

Psychological profiling also revealed distinct patterns. *Candida*-positive patients with primary BMS demonstrated higher distress scores in somatization, anxiety, and psychoticism, whereas *Candida*-positive patients in the total sample and overall BMS subgroup demonstrated higher phobic anxiety and psychoticism scores. The high prevalence of psychological distress in BMS—including depression and anxiety—is well established [39, 40]. Beyond this, recent evidence suggests that psychological distress-related neuroendocrine–immune pathways can modulate oral microbial ecology, impair salivary gland function and alter mucosal immunity [41]. Experimental models confirm that psychological stress promotes oral *Candida albicans* colonization and hyphal invasion, effects reversed by anxiolytic treatment [42]. Clinical studies in psychiatric populations similarly report oral fungal dysbiosis, including *Candida* overgrowth, linked to systemic immune dysregulation and heightened pro-inflammatory signaling [43].

In BMS, altered bacterial microbiota and salivary metabolites have been linked to higher anxiety and depression scores, and distinct oral microbiome profiles have been identified in BMS patients without salivary hypofunction, suggesting that microbial alterations may be intrinsic to the disorder [44, 45]. Collectively, these findings support a bidirectional model in which psychological distress increases susceptibility to *Candida* colonization, while *Candida* infection, within a peripherally and centrally sensitized trigeminal–gustatory network, further amplifies sensory disturbances and psychological burden. This perspective aligns with a biopsychosocial framework for BMS and taste disorders, in which microbial, neural, and psychological factors interact synergistically to shape clinical outcomes.

Our results highlight the importance of considering positive oral *Candida* culture as an independent factor in patients with taste disturbances, rather than automatically categorizing it as a cause of secondary BMS. This approach avoids misclassification and enables more targeted interventions—such as antifungal therapy, salivary stimulation, or psychosocial support—particularly for older adults with reduced salivary flow. Clinicians should recognize that *Candida*-related taste changes may be subtle and detectable only through objective testing.

However, this study has several limitations. First, the retrospective design limits causal inference, and unmeasured confounding cannot be excluded. Second, microbiological analysis relied on culture-based *Candida* detection without precise quantification, species-level identification, or assessment of strain virulence. Third, despite age-matching, other potential confounders, including medication use and systemic comorbidities, were not fully controlled. Moreover, the cohort was predominantly female and consisted mainly of middle-to-older adults, which may limit the generalizability of the findings to male patients or younger populations. Finally, the cross-sectional gustatory testing precludes determining whether *Candida* colonization precedes or follows taste alterations. Future studies incorporating molecular microbiome profiling, quantitative salivary biomarker diagnostics, and multiple standardized gustatory testing methods could provide more definitive evidence. Randomized controlled trials of antifungal therapy in well-stratified patient populations would be particularly valuable in clarifying the causal role of *Candida* in taste disturbances and burning mouth symptoms.

## Conclusions

This study demonstrates that positive oral *Candida* culture is not universally associated with impaired taste function but may contribute to subtle gustatory deficits and reduced stimulated salivary flow in specific patient subgroups, particularly in primary BMS. Psychological distress was also more pronounced in certain *Candida*-positive subgroups, emphasizing the complex interplay between microbial, physiological, and psychosocial factors in oral symptomatology. By strategically excluding *Candida* culture-positive status from the definition of secondary BMS, we evaluated its independent impact, providing evidence to support more precise diagnostic classification and tailored management. These findings highlight the value of an integrated clinical approach that combines microbiological, salivary, gustatory, and psychological evaluations in patients with taste disturbances, particularly older adults who are at increased risk of salivary hypofunction and oral *Candida* colonization.

## Supplementary Information

Below is the link to the electronic supplementary material.

Supplementary Material 1 (DOCX 75.4 KB)

## Data Availability

The data that support the findings of this study are available from the corresponding author upon reasonable request.
