# Supplementary material for: Clinical and gustatory features in taste disorder patients based on oral Candida culture status
Source: Clin Oral Investig. 2026 Mar 13;30(4):122. doi: 10.1007/s00784-026-06816-9 (PMC12987823; doi:10.1007/s00784-026-06816-9)
Supplement: Supplementary file 1 — Supplementary Material 1 (DOCX 75.4 KB) [file 784_2026_6816_MOESM1_ESM.docx]

**Supplementary Table 1-1. Demographic characteristics and salivary flow rates based on oral *Candida* culture status in the total sample and patients with burning mouth syndrome; mean ± SD, median [Q1–Q3], *n* (%).**

|  | **Total sample (*n* = 131)** | |  | **BMS (+) (*n* = 94)** | |  |
| --- | --- | --- | --- | --- | --- | --- |
|  | ***C* (−) (*n* = 98)** | ***C* (+) (*n* = 33)** | ***p*** | ***C* (−) (*n* = 71)** | ***C* (+) (*n* = 23)** | ***p*** |
| Age (years) | 61.3 ± 11.4 | 68.2 ± 9.5 | 0.002** | 61.5 ± 10.3 | 67.9 ± 9.7 | 0.010* |
| Gender |  |  |  |  |  |  |
| Male | 19 (19.4) | 6 (18.2) | 1.000 | 7 (9.9) | 3 (13.0) | 0.702 |
| Female | 79 (80.6) | 27 (81.8) |  | 64 (90.1) | 20 (87.0) |  |
| SFR (mL/min) |  |  |  |  |  |  |
| UWS | 0.20 [0.10−0.33] | 0.15 [0.06−0.25] | 0.060 | 0.20 [0.18−0.33] | 0.15 [0.06−0.21] | 0.085 |
| SWS | 1.21 [0.70−1.67]^a^ | 0.91 [0.70−1.16]^a^ | 0.061 | 1.22 [0.71−1.64] | 0.94 [0.77−1.37]^a^ | 0.445 |

^a^ Stimulated whole saliva could not be collected from seven patients (one oral *Candida* (−) and six oral *Candida* (+)), including four with BMS, due to the inability to chew paraffin wax caused by missing posterior teeth.

*P*-values were calculated using the Student’s *t*-test or Mann–Whitney *U* test for continuous variables, as appropriate, and the Fisher’s exact test for gender comparisons.

Abbreviations: BMS, burning mouth syndrome; *C* (−): oral *Candida* culture negative; *C* (+): oral *Candida* culture positive; Q1, 25th percentile; Q3, 75th percentile; SD, standard deviation; SFR, salivary flow rate, SWS, stimulated whole saliva; UWS, unstimulated whole saliva.

* *p* < 0.05, ** *p* < 0.01

**Supplementary Table 1-2. Demographic characteristics and salivary flow rates based on oral *Candida* culture status in age-matched patients; mean ± SD, median [Q1–Q3], *n* (%).**

|  | **Primary BMS – age matched** | |  | **Total sample – age matched** | |  | **BMS (+) – age matched** | |  |
| --- | --- | --- | --- | --- | --- | --- | --- | --- | --- |
|  | ***C* (−) (*n* = 23)** | ***C* (+) (*n* = 11)** | ***p*** | ***C* (−) (*n* = 52)** | ***C* (+) (*n* = 33)** | ***p*** | ***C* (−) (*n* = 37)** | ***C* (+) (*n* = 23)** | ***p*** |
| Age (years) | 67.5 ± 6.7 | 68.3 ± 8.2 | 0.506 | 69.8 ± 5.8 | 68.2 ± 9.5 | 0.807 | 69.7 ± 6.1 | 67.9 ± 9.7 | 0.861 |
| Gender |  |  |  |  |  |  |  |  |  |
| Male | 2 (8.7) | 1 (9.1) | 1.000 | 10 (19.2) | 6 (18.2) | 0.904 | 4 (10.8) | 3 (13.0) | 1.000 |
| Female | 21 (91.3) | 10 (90.9) |  | 42 (80.8) | 27 (81.8) |  | 33 (89.2) | 20 (87.0) |  |
| SFR (mL/min) |  |  |  |  |  |  |  |  |  |
| UWS | 0.26 [0.15–0.37] | 0.15 [0.07–0.27] | 0.293 | 0.17 [0.08–0.30] | 0.15 [0.06–0.25] | 0.386 | 0.17 [0.07–0.33] | 0.15 [0.06–0.21] | 0.392 |
| SWS | 1.22 [0.71–1.51] | 0.81 [0.73–0.97]^a^ | 0.043* | 1.10 [0.64–1.55]^a^ | 0.91 [0.70–1.16]^a^ | 0.361 | 1.10 [0.65–1.46] | 0.94 [0.77–1.37]^a^ | 0.842 |

*Note*: Age matching was performed by selecting the older half of patients in the *Candida* (−) group in cases with significant age differences between the *Candida* (−) and *Candida* (+) groups.

^a^ Stimulated whole saliva could not be collected from seven patients in the age-matched groups (one oral *Candida* (−) and six oral *Candida* (+)), including two with primary BMS, and four with BMS due to the inability to chew paraffin wax caused by missing posterior teeth.

*P*-values were calculated using the Student’s *t*-test or Mann–Whitney *U* test for continuous variables, as appropriate, and the Fisher’s exact test for gender comparisons.

Abbreviations: BMS, burning mouth syndrome; *C* (−): oral *Candida* culture negative; *C* (+): oral *Candida* culture positive; Q1, 25th percentile; Q3, 75th percentile; SD, standard deviation; SFR, salivary flow rate, SWS, stimulated whole saliva; UWS, unstimulated whole saliva.

* *p* < 0.05

**Supplementary Table 2-1. Subjective and objective taste scores based on oral *Candida* culture status in the total sample and patients with burning mouth syndrome; median [Q1–Q3].**

|  | **Total sample (*n* = 131)** | |  | **BMS (+) (*n* = 94)** | |  |
| --- | --- | --- | --- | --- | --- | --- |
|  | ***C* (−) (*n* = 98)** | ***C* (+) (*n* = 33)** | ***p*** | ***C* (−) (*n* = 71)** | ***C* (+) (*n* = 23)** | ***p*** |
| Subjective taste |  |  |  |  |  |  |
| Sweet | 2.0 [1.0–2.0] | 2.0 [1.0–2.0] | 0.546 | 2.0 [1.0–2.0] | 2.0 [1.0–2.0] | 0.868 |
| Sour | 2.0 [1.0–2.0] | 2.0 [2.0–2.0] | 0.380 | 2.0 [1.0–2.0] | 2.0 [2.0–2.0] | 0.460 |
| Salty | 2.0 [1.0–2.0] | 2.0 [1.0–2.0] | 0.589 | 2.0 [1.0–2.0] | 2.0 [1.0–2.0] | 0.746 |
| Bitter | 2.0 [1.0–2.0] | 2.0 [1.0–2.0] | 0.176 | 2.0 [1.0–2.0] | 2.0 [1.0–2.0] | 0.296 |
| Total | 8.0 [4.8–8.0] | 7.0 [5.3–8.0] | 0.598 | 8.0 [4.0–8.0] | 7.0 [5.0–8.0] | 0.406 |
| Objective taste |  |  |  |  |  |  |
| Sweet | 3.0 [2.0–4.0] | 4.0 [2.5–4.0] | 0.603 | 4.0 [2.0–4.0] | 4.0 [3.0–4.0] | 0.587 |
| Sour | 2.0 [1.0–3.0] | 2.0 [0.5–3.0] | 0.276 | 2.0 [1.0–3.0] | 2.0 [1.0–3.0] | 0.764 |
| Salty | 3.0 [2.0–4.0] | 3.0 [1.0–3.0] | 0.941 | 3.0 [2.0–4.0] | 3.0 [1.0–3.0] | 0.274 |
| Bitter | 3.0 [2.0–4.0] | 3.0 [0.5–4.0] | 0.404 | 3.0 [2.0–4.0] | 3.0 [0.0–4.0] | 0.374 |
| Total | 10.0 [7.8–12.3] | 10.0 [7.0–12.0] | 0.421 | 11.0 [8.0–13.0] | 10.0 [8.0–12.0] | 0.346 |

*Note*: Subjective taste scores were obtained from a structured questionnaire in which patients rated their ability to detect each taste quality on a scale ranging from 0 (not at all) to 2 (easily), yielding a maximum total score of 8. Objective taste scores were obtained using filter paper taste strips (*Taste Strips*; Burghart Messtechnik, Holm, Pinneberg, Germany), with each taste quality (sweet, salty, sour, and bitter) scored 0–4 based on correct identifications (maximum total score 16).

*P*-values were calculated using the Student’s *t*-test or the Mann–Whitney *U* test, as appropriate, for comparisons between *Candida* (+) and *Candida* (−) within each group.

Abbreviations: BMS, burning mouth syndrome; *C* (−): oral *Candida* culture negative; *C* (+): oral *Candida* culture positive; Q1, 25th percentile; Q3, 75th percentile.

**Supplementary Table 2-2. Subjective and objective taste scores based on oral *Candida* culture status in age-matched patients; median [Q1–Q3].**

|  | **Primary BMS – age matched** | |  | **Total sample – age matched** | |  | **BMS (+) – age matched** | |  |
| --- | --- | --- | --- | --- | --- | --- | --- | --- | --- |
|  | ***C* (−) (*n* = 23)** | ***C* (+) (*n* = 11)** | ***p*** | ***C* (−) (*n* = 52)** | ***C* (+) (*n* = 33)** | ***p*** | ***C* (−) (*n* = 37)** | ***C* (+) (*n* = 23)** | ***p*** |
| Subjective taste |  |  |  |  |  |  |  |  |  |
| Sweet | 1.0 [1.0–2.0] | 2.0 [1.0–2.0] | 0.148 | 2.0 [1.0–2.0] | 2.0 [1.0–2.0] | 0.382 | 2.0 [1.0–2.0] | 2.0 [1.0–2.0] | 0.217 |
| Sour | 2.0 [1.0–2.0] | 2.0 [2.0–2.0] | 0.074 | 2.0 [1.0–2.0] | 2.0 [2.0–2.0] | 0.176 | 2.0 [1.0–2.0] | 2.0 [2.0–2.0] | 0.111 |
| Salty | 2.0 [1.0–2.0] | 2.0 [1.0–2.0] | 0.638 | 2.0 [1.0–2.0] | 2.0 [1.0–2.0] | 0.280 | 2.0 [1.0–2.0] | 2.0 [1.0–2.0] | 0.525 |
| Bitter | 2.0 [1.0–2.0] | 2.0 [1.0–2.0] | 0.581 | 2.0 [1.0–2.0] | 2.0 [1.0–2.0] | 0.628 | 2.0 [1.0–2.0] | 2.0 [1.0–2.0] | 0.959 |
| Total | 7.0 [4.0–8.0] | 7.0 [7.0–8.0] | 0.440 | 8.0 [4.0–8.0] | 7.0 [5.3–8.0] | 0.843 | 7.0 [4.0–8.0] | 7.0 [5.0–8.0] | 0.587 |
| Objective taste |  |  |  |  |  |  |  |  |  |
| Sweet | 4.0 [3.0–4.0] | 4.0 [3.0–4.0] | 0.869 | 3.0 [2.0–4.0] | 4.0 [2.5–4.0] | 0.772 | 4.0 [2.5–4.0] | 4.0 [3.0–4.0] | 0.582 |
| Sour | 2.0 [2.0–3.0] | 1.0 [1.0–2.0] | 0.173 | 2.0 [1.0–3.0] | 2.0 [0.5–3.0] | 0.422 | 2.0 [1.0–3.0] | 2.0 [1.0–3.0] | 0.844 |
| Salty | 3.0 [2.0–4.0] | 3.0 [3.0–3.0] | 0.801 | 3.0 [1.0–4.0] | 3.0 [1.0–3.0] | 0.889 | 3.0 [1.5–4.0] | 3.0 [1.0–3.0] | 0.282 |
| Bitter | 3.0 [2.0–4.0] | 3.0 [0.0–3.0] | 0.096 | 3.0 [2.0–4.0] | 3.0 [0.5–4.0] | 0.546 | 3.0 [2.0–4.0] | 3.0 [0.0–4.0] | 0.415 |
| Total | 12.0 [10.0–13.0] | 10.0 [8.0–12.0] | 0.049* | 10.0 [7.3–12.0] | 10.0 [7.0–12.0] | 0.546 | 10.0 [8.5–12.5] | 10.0 [8.0–12.0] | 0.559 |

*Note*: Subjective taste scores were obtained from a structured questionnaire in which patients rated their ability to detect each taste quality on a scale ranging from 0 (not at all) to 2 (easily), yielding a maximum total score of 8. Objective taste scores were obtained using filter paper taste strips (*Taste Strips*; Burghart Messtechnik, Holm, Pinneberg, Germany), with each taste quality (sweet, salty, sour, and bitter) scored 0–4 based on correct identifications (maximum total score 16).

Age matching was performed by selecting the older half of patients in the *Candida* (−) group in cases with significant age differences between the *Candida* (−) and *Candida* (+) groups.

*P*-values were calculated using the Student’s *t*-test or the Mann–Whitney *U* test, as appropriate, for comparisons between *Candida* (+) and *Candida* (−) within each group.

Abbreviations: BMS, burning mouth syndrome; *C* (−): oral *Candida* culture negative; *C* (+): oral *Candida* culture positive; Q1, 25th percentile; Q3, 75th percentile.

* *p* < 0.05

**Supplementary Table 3-1. Distribution of subjective and objective taste diagnoses based on oral *Candida* culture status in the total sample and patients with burning mouth syndrome; *n* (%).**

|  | **Total sample (*n* = 131)** | |  | **BMS (+) (*n* = 94)** | |  |
| --- | --- | --- | --- | --- | --- | --- |
|  | ***C* (−) (*n* = 98)** | ***C* (+) (*n* = 33)** | ***p*** | ***C* (−) (*n* = 71)** | ***C* (+) (*n* = 23)** | ***p*** |
| Subjective taste |  |  |  |  |  |  |
| Normogeusia | 11 (11.2) | 6 (18.2) | 0.227 | 9 (12.7) | 4 (17.4) | 0.428 |
| Hypogeusia | 37 (37.8) | 18 (54.5) |  | 24 (33.8) | 12 (52.2) |  |
| Dysgeusia | 19 (19.4) | 4 (12.1) |  | 14 (19.7) | 2 (8.7) |  |
| Hypo + Dysgeusia | 25 (25.5) | 4 (12.1) |  | 20 (28.2) | 4 (17.4) |  |
| Ageusia | 6 (6.1) | 1 (3.0) |  | 4 (5.6) | 1 (4.3) |  |
| Objective taste |  |  |  |  |  |  |
| Normogeusia | 27 (27.6) | 4 (12.1) | 0.208 | 23 (32.4) | 3 (13.0) | 0.109 |
| Hypogeusia | 7 (7.1) | 1 (3.0) |  | 5 (7.0) | 0 (0.0) |  |
| Dysgeusia | 38 (38.8) | 16 (48.5) |  | 29 (40.8) | 12 (52.2) |  |
| Hypo + Dysgeusia | 26 (26.5) | 12 (36.4) |  | 14 (19.7) | 8 (34.8) |  |

*Note*: Subjective taste diagnoses were based on questionnaire responses: normogeusia was defined as normal taste perception; hypogeusia as self-reported decreased taste; dysgeusia as distorted, exaggerated, or phantom taste sensations; and ageusia as complete taste loss. Objective taste diagnoses were based on taste strip test results: normogeusia was defined as a total score ≥ 9; hypogeusia as a total score < 9; dysgeusia as incorrect responses at the strongest or second-strongest concentration for any taste quality. Hypo + dysgeusia indicates patients meeting both criteria.

*P*-values were calculated using the Chi-square test or the Fisher’s exact test, as appropriate, for comparisons between oral *Candida* (−) and oral *Candida* (+) within each group.

Abbreviations: BMS, burning mouth syndrome; *C* (−), oral *Candida* culture negative; *C* (+), oral *Candida* culture positive.

**Supplementary Table 3-2. Distribution of subjective and objective taste diagnoses based on oral *Candida* culture status in age-matched patients; *n* (%).**

|  | **Primary BMS – age matched** | |  | **Total sample – age matched** | |  | **BMS (+) – age matched** | |  |
| --- | --- | --- | --- | --- | --- | --- | --- | --- | --- |
|  | ***C* (−) (*n* = 23)** | ***C* (+) (*n* = 11)** | ***p*** | ***C* (−) (*n* = 52)** | ***C* (+) (*n* = 33)** | ***p*** | ***C* (−) (*n* = 37)** | ***C* (+) (*n* = 23)** | ***p*** |
| Subjective taste |  |  |  |  |  |  |  |  |  |
| Normogeusia | 3 (13.0) | 2 (18.2) | 0.974 | 3 (5.8) | 6 (18.2) | 0.256 | 3 (8.1) | 4 (17.4) | 0.792 |
| Hypogeusia | 10 (43.5) | 6 (54.5) |  | 25 (48.1) | 18 (54.5) |  | 17 (45.9) | 12 (52.2) |  |
| Dysgeusia | 3 (13.0) | 1 (9.1) |  | 7 (13.5) | 4 (12.1) |  | 5 (13.5) | 2 (8.7) |  |
| Hypo + Dysgeusia | 5 (21.7) | 2 (18.2) |  | 13 (25.0) | 4 (12.1) |  | 9 (24.3) | 4 (17.4) |  |
| Ageusia | 2 (8.7) | 0 (0.0) |  | 4 (7.7) | 1 (3.0) |  | 3 (8.1) | 1 (4.3) |  |
| Objective taste |  |  |  |  |  |  |  |  |  |
| Normogeusia | 9 (39.1) | 0 (0.0) | 0.008** | 13 (25.0) | 4 (12.1) | 0.517 | 11 (29.7) | 3 (13.0) | 0.233 |
| Hypogeusia | 1 (4.3) | 0 (0.0) |  | 2 (3.8) | 1 (3.0) |  | 2 (5.4) | 0 (0.0) |  |
| Dysgeusia | 12 (52.2) | 7 (63.6) |  | 22 (42.3) | 16 (48.5) |  | 17 (45.9) | 12 (52.2) |  |
| Hypo + Dysgeusia | 1 (4.3) | 4 (36.4) |  | 15 (28.8) | 12 (36.4) |  | 7 (18.9) | 8 (34.8) |  |

*Note*: Subjective taste diagnoses were based on questionnaire responses: normogeusia was defined as normal taste perception; hypogeusia as self-reported decreased taste; dysgeusia as distorted, exaggerated, or phantom taste sensations; and ageusia as complete taste loss. Objective taste diagnoses were based on taste strip test results: normogeusia was defined as a total score ≥ 9; hypogeusia as a total score < 9; dysgeusia as incorrect responses at the strongest or second-strongest concentration for any taste quality. Hypo + dysgeusia indicates patients meeting both criteria.

Age matching was performed by selecting the older half of patients in the *Candida* (−) group in cases with significant age differences between the *Candida* (−) and *Candida* (+) groups.

*P*-values were calculated using the Chi-square test or the Fisher’s exact test, as appropriate, for comparisons between oral *Candida* (−) and oral *Candida* (+) within each group.

Abbreviations: BM, burning mouth; BMS, burning mouth syndrome; *C* (−), oral *Candida* culture negative; *C* (+), oral *Candida* culture positive.

** *p* < 0.01

**Supplementary Table 4. Intensities of burning mouth symptom based on oral *Candida* culture status in age-matched patients; median [Q1–Q3].**

|  | | **Primary BMS – age matched** | |  | **BMS (+) – age matched** | |  |
| --- | --- | --- | --- | --- | --- | --- | --- |
| **Intensity (VAS)** | | ***C* (−) (*n* = 23)** | ***C* (+) (*n* = 11)** | ***p*** | ***C* (−) (*n* = 37)** | ***C* (+) (*n* = 23)** | ***p*** |
| Burning | AVG | 5.0 [2.0–5.0] | 5.0 [3.0–7.0] | 0.151 | 5.0 [2.0–6.5] | 5.0 [3.0–7.0] | 0.770 |
|  | MAX | 6.0 [5.0–8.0] | 8.0 [6.0–8.0] | 0.140 | 7.0 [4.3–8.0] | 7.0 [5.0–8.0] | 0.578 |
| Aching | AVG | 0.0 [0.0–5.0] | 5.0 [0.0–6.0] | 0.051 | 0.0 [0.0–6.0] | 3.0 [0.0–5.0] | 0.625 |
|  | MAX | 0.0 [0.0–6.0] | 7.0 [0.0–8.0] | 0.066 | 0.0 [0.0–7.5] | 6.0 [0.0–8.0] | 0.492 |
| Stinging | AVG | 0.0 [0.0–1.0] | 2.0 [0.0–7.0] | 0.037* | 0.0 [0.0–3.5] | 1.0 [0.0–5.0] | 0.582 |
|  | MAX | 0.0 [0.0–2.0] | 7.0 [0.0–8.0] | 0.030* | 0.0 [0.0–5.0] | 3.0 [0.0–8.0] | 0.298 |
| Numbness | AVG | 0.0 [0.0–3.0] | 1.0 [0.0–5.0] | 0.208 | 0.0 [0.0–2.0] | 0.0 [0.0–5.0] | 0.146 |
|  | MAX | 0.0 [0.0–4.0] | 1.0 [0.0–8.0] | 0.200 | 0.0 [0.0–3.0] | 0.0 [0.0–7.0] | 0.135 |
| Taste−dist | AVG | 5.0 [1.0–7.0] | 7.0 [3.0–7.5] | 0.601 | 6.0 [3.5–8.0] | 6.0 [4.0–7.5] | 0.713 |
|  | MAX | 7.0 [2.0–9.0] | 8.0 [6.0–8.0] | 0.562 | 8.0 [5.0–9.0] | 8.0 [6.0–9.0] | 0.890 |
| Xerostomia | AVG | 2.0 [0.0–7.0] | 5.0 [3.0–7.0] | 0.340 | 3.0 [0.0–6.5] | 5.0 [3.0–7.0] | 0.267 |
|  | MAX | 5.0 [1.0–8.0] | 8.0 [3.0–9.0] | 0.163 | 5.0 [0.5–8.0] | 7.0 [5.0–9.0] | 0.930 |
| Eff−life | AVG | 5.0 [2.0–7.0] | 5.0 [4.0–8.0] | 0.649 | 5.0 [3.0–8.0] | 5.0 [3.0–8.0] | 0.612 |
|  | MAX | 7.0 [2.0–9.0] | 8.0 [5.0–9.0] | 0.469 | 8.0 [5.0–9.0] | 8.0 [5.0–9.0] | 1.000 |

*Note:* Symptom intensities were assessed using visual analogue scale (VAS) scores ranging from 0 (no symptom) to 10 (most extreme symptom imaginable). AVG and MAX represent average and maximum scores, respectively, for each symptom domain.
*P*-values were calculated using the Mann–Whitney *U* test for comparisons between *Candida* (+) and *Candida* (−) within each group.

Abbreviations**:** AVG, average; BMS, burning mouth syndrome; *C* (−), oral *Candida* culture negative; *C* (+), oral *Candida* culture positive; Eff-life, effect of symptoms on daily life; MAX, maximum; Q1, 25th percentile; Q3, 75th percentile; Taste-dist, taste disturbance; VAS, visual analogue scale.

* *p* < 0.05

**Supplementary Table 5-1. Psychological profiles based on oral *Candida* culture status in the total sample and patients with burning mouth syndrome; median [Q1–Q3].**

|  | **Total sample (*n* = 118)^a^** | |  | **BMS (+) (*n* = 91)^a^** | |  |
| --- | --- | --- | --- | --- | --- | --- |
| *t*-score | ***C* (−) (*n* = 88)** | ***C* (+) (*n* = 30)** | ***p*** | ***C* (−) (*n* = 69)** | ***C* (+) (*n* = 22)** | ***p*** |
| Somatization | 44.0 [38.3–51.0] | 45.0 [43.0–51.0] | 0.083 | 44.0 [40.0–51.0] | 47.5 [44.8–51.5] | 0.145 |
| O-C | 42.0 [36.0–46.0] | 42.5 [39.5–48.8] | 0.117 | 42.0 [36.0–46.5] | 44.0 [39.5–51.5] | 0.067 |
| I-S | 41.0 [36.0–44.8] | 43.0 [39.0–49.3] | 0.068 | 43.0 [36.5–46.5] | 43.5 [40.5–52.3] | 0.118 |
| Depression | 44.5 [39.0–49.8] | 45.5 [41.0–53.0] | 0.261 | 45.0 [40.0–50.0] | 45.5 [41.8–53.0] | 0.383 |
| Anxiety | 42.0 [38.3–46.0] | 43.0 [39.8–46.8] | 0.394 | 43.0 [39.0–46.0] | 43.5 [39.8–46.8] | 0.385 |
| Hostility | 42.0 [40.0–45.0] | 43.0 [40.0–47.3] | 0.326 | 43.0 [40.0–45.0] | 43.0 [39.5–48.0] | 0.239 |
| Phobic anxiety | 42.0 [40.0–45.0] | 43.0 [41.5–48.0] | 0.026* | 42.0 [40.0–45.0] | 43.0 [40.0–49.3] | 0.045* |
| Paranoid ideation | 40.0 [38.0–42.0] | 40.0 [38.0–48.0] | 0.191 | 40.0 [38.0–42.0] | 40.0 [38.0–48.0] | 0.235 |
| Psychoticism | 42.0 [39.0–45.8] | 45.0 [41.0–50.0] | 0.015* | 43.0 [39.0–46.0] | 45.0 [41.0–50.0] | 0.029* |
| GSI | 42.0 [38.0–47.0] | 43.0 [39.8–49.3] | 0.129 | 43.0 [38.5–48.0] | 43.5 [41.0–50.5] | 0.069 |
| PSDI | 43.0 [39.0–50.0] | 42.0 [39.0–48.0] | 0.473 | 43.0 [40.0–50.0] | 41.0 [39.0–49.0] | 0.428 |
| PST | 42.5 [35.3–48.0] | 46.0 [40.0–57.0] | 0.025* | 44.0 [37.0–49.0] | 47.0 [41.8–57.3] | 0.021* |

*Note*: Psychological profile scores are presented as *t*-scores from the Symptom Checklist-90-Revised (SCL-90-R), comprising nine symptom dimensions and three global indices.

^a^ SCL-90-R data were available for 118 of 131 participants in the total sample, including 91 of 94 patients with BM symptoms.

*P*-values were calculated using the Student’s *t*-test or the Mann–Whitney *U* test, as appropriate, for comparisons between *Candida* (+) and *Candida* (−) within each group.

Abbreviations: BM, burning mouth; BMS, burning mouth syndrome; *C* (−), oral *Candida* culture negative; *C* (+), oral *Candida* culture positive; GSI, global severity index; I-S, interpersonal sensitivity; O-C, obsessive compulsive; PSDI, positive symptom distress index; PST, positive symptom total; Q1, 25th percentile; Q3, 75th percentile.

* *p* < 0.05

**Supplementary Table 5-2. Psychological profiles based on oral *Candida* culture status in age-matched patients; median [Q1–Q3].**

|  | **Primary BMS – age matched** | |  | **Total sample – age matched^a^** | |  | **BMS (+) – age matched^a^** | |  |
| --- | --- | --- | --- | --- | --- | --- | --- | --- | --- |
| *t*-score | ***C* (−) (*n* = 23)** | ***C* (+) (*n* = 11)** | ***p*** | ***C* (−) (*n* = 48)** | ***C* (+) (*n* = 30)** | ***p*** | ***C* (−) (*n* = 36)** | ***C* (+) (*n* = 22)** | ***p*** |
| Somatization | 44.0 [38.0–53.0] | 48.0 [45.0–51.0] | 0.160 | 44.0 [38.0–52.0] | 45.0 [43.0–51.0] | 0.321 | 47.0 [38.5–53.0] | 47.5 [44.8–51.5] | 0.441 |
| O-C | 42.0 [34.0–46.0] | 45.0 [40.0–51.0] | 0.182 | 42.0 [35.0–46.0] | 42.5 [39.5–48.8] | 0.085 | 42.0 [35.0–46.0] | 44.0 [39.5–51.5] | 0.090 |
| I-S | 41.0 [36.0–48.0] | 43.0 [41.0–52.0] | 0.182 | 40.5 [36.0–44.0] | 43.0 [39.0–49.3] | 0.036* | 42.0 [36.0–44.8] | 43.5 [40.5–52.3] | 0.079 |
| Depression | 45.0 [37.0–50.0] | 46.0 [42.0–53.0] | 0.329 | 44.5 [37.3–51.8] | 45.5 [41.0–53.0] | 0.420 | 47.5 [38.3–52.8] | 45.5 [41.8–53.0] | 0.648 |
| Anxiety | 41.0 [38.0–46.0] | 44.0 [42.0–46.0] | 0.271 | 42.0 [38.0–47.8] | 43.0 [39.8–46.8] | 0.487 | 43.0 [38.3–49.0] | 43.5 [39.8–46.8] | 0.641 |
| Hostility | 43.0 [38.0–48.0] | 45.0 [42.0–48.0] | 0.280 | 43.0 [38.5–45.0] | 43.0 [40.0–47.3] | 0.543 | 43.0 [40.0–47.3] | 43.0 [39.5–48.0] | 0.496 |
| Phobic anxiety | 42.0 [40.0–45.0] | 43.0 [40.0–48.0] | 0.543 | 42.0 [40.0–45.0] | 43.0 [41.5–48.0] | 0.068 | 42.0 [40.0–45.0] | 43.0 [40.0–49.3] | 0.144 |
| Paranoid ideation | 40.0 [38.0–42.0] | 40.0 [38.0–51.0] | 0.346 | 40.0 [38.0–42.0] | 40.0 [38.0–48.0] | 0.166 | 40.0 [38.0–42.0] | 40.0 [38.0–48.0] | 0.197 |
| Psychoticism | 41.0 [39.0–46.0] | 45.0 [41.0–50.0] | 0.099 | 42.0 [39.0–46.0] | 45.0 [41.0–50.0] | 0.048* | 43.0 [39.0–47.5] | 45.0 [41.0–50.0] | 0.118 |
| GSI | 43.0 [36.0–50.0] | 44.0 [42.0–50.0] | 0.315 | 42.5 [37.3–49.0] | 43.0 [39.8–49.3] | 0.267 | 44.0 [37.8–50.0] | 43.5 [41.0–50.5] | 0.253 |
| PSDI | 41.0 [39.0–50.0] | 41.0 [39.0–48.0] | 0.985 | 45.0 [39.5–52.0] | 42.0 [39.0–48.0] | 0.233 | 45.5 [41.0–54.0] | 41.0 [39.0–49.0] | 0.154 |
| PST | 42.0 [33.0–50.0] | 48.0 [42.0–57.0] | 0.119 | 42.0 [34.3–48.0] | 46.0 [40.0–57.0] | 0.037* | 43.0 [34.8–48.8] | 47.0 [41.8–57.3] | 0.040* |

*Note*: Psychological profile scores are presented as *t*-scores from the Symptom Checklist-90-Revised (SCL-90-R), comprising nine symptom dimensions and three global indices.

^a^ SCL-90-R data were available for 78 of 85 patients in the age-matched total sample, including 58 of 60 patients with BM symptoms.

Age matching was performed by selecting the older half of patients in the *Candida* (−) group in cases with significant age differences between the *Candida* (−) and *Candida* (+) groups.

*P*-values were calculated using the Student’s *t*-test or the Mann–Whitney *U* test, as appropriate, for comparisons between *Candida* (+) and *Candida* (−) within each group.

Abbreviations: BM, burning mouth; BMS, burning mouth syndrome; *C* (−), oral *Candida* culture negative; *C* (+), oral *Candida* culture positive; GSI, global severity index; I-S, interpersonal sensitivity; O-C, obsessive compulsive; PSDI, positive symptom distress index; PST, positive symptom total; Q1, 25th percentile; Q3, 75th percentile.

* *p* < 0.05
